# Stratified Use of Genetic Testing in Liver Disease Improves Diagnostic Yield and Clinical Impact

**DOI:** 10.3390/ijms27177906

**Published:** 2026-09-04

**Authors:** Angelo Corso Faini, Michele Pinon, Giulia Margherita Brach Del Prever, Carmelo Maria Romeo, Maria Luca, Fiorenza Mioli, Claudia Saglia, Caterina Scolari, Tullia Carradori, Francesca Arruga, Cristina Chiadò, Anna Morgando, Silvia Martini, Renato Romagnoli, Elisabetta Bugianesi, Diana Carli, Tiziana Vaisitti, Silvia Deaglio, Pier Luigi Calvo

**Affiliations:** 1 Immunogenetics and Transplant Biology Service, AOU Città della Salute e della Scienza Hospital, 10126 Turin, Italy; 2 Department of Medical Sciences, University of Turin, 10126 Turin, Italy; 3 Pediatric Gastroenterology Division, Regina Margherita Children’s Hospital, 10126 Turin, Italy; 4 Division of Gastroenterology, AOU Città della Salute e della Scienza Hospital, 10126 Turin, Italy; 5 SSD Insufficienza Epatica e Trapianto Epatico, AOU Città della Salute e della Scienza Hospital, 10126 Turin, Italy; 6 General Surgery 2U and Liver Transplantation Center, AOU Città della Salute e della Scienza Hospital, 10126 Turin, Italy

**Keywords:** high-throughput nucleotide sequencing, liver diseases, genetic testing

## Abstract

Genetic testing plays an important role in the diagnosing of liver diseases, but its diagnostic performance varies across patient populations. By analyzing diagnostic yield across patient subgroups, this study aims at defining age-tailored diagnostic workflows for a more effective integration of genetic testing into clinical practice. We retrospectively analyzed 203 patients (145 children, 58 adults) with acute or subacute liver disorders of suspected genetic origin who underwent next-generation sequencing, categorized them by clinical diagnosis, and evaluated diagnostic yields to develop age-tailored testing workflows. The median age was 8 years; 65.5% were male, 84.7% White, and 27.6% had undergone liver transplantation. Cholestatic liver disorders (30%) and unexplained liver dysfunction (20.2%) were the most common indications for testing. Overall, genetic testing achieved a definitive diagnosis in 35.5% of patients, with a higher yield in children than adults (41.4% vs. 20.7%). Metabolic disorders had the highest diagnostic yield (83.3%), while PFIC/BRIC and Alagille syndrome were the most frequent genetic diagnoses. Age-specific diagnostic workflows retrospectively enriched the overall diagnostic rate by approximately 11% across age and disease categories. These findings demonstrate that tailoring genetic testing strategies to patient age and clinical presentation can improve diagnostic efficiency and support a standardized integration of genetic testing into hepatology practice.

## 1. Introduction

Liver disease is a major health issue, causing 2 million deaths worldwide each year and accounting for ≈4% of total annual deaths [1]. Under the broad definition of liver diseases, multiple subcategories can be identified: in the adult population and in order of prevalence, Metabolic dysfunction-Associated Steatotic Liver Disease (MASLD, 30% of the global population [2]), alcohol-related liver disease (ALD) [1], and autoimmune and viral B and C hepatitis top the list [3]. Genetically determined disorders represent a minor proportion of all adult liver diseases, mainly belonging to the rare conditions category [4]. On the contrary, in the pediatric population, liver diseases often arise from genetic conditions, which can present with a diverse spectrum of manifestations, ranging from acute and potentially fatal liver failure to relatively asymptomatic cases. Even more strikingly, it is estimated that 50% of chronic liver diseases and 20% of liver transplants (LT) have an underlying genetic disorder [5].

When a genetic condition is suspected, traditional diagnostic approaches including liver biopsies, blood tests and imaging may fail to define or significantly delay diagnosis, particularly in cases where phenotypic overlap exists between multiple potential disorders. In fact, many genetic liver conditions may present with subtle or non-specific symptoms that can be overlooked or misinterpreted, potentially preventing early intervention, which is crucial for the management of some liver conditions.

In this context, genetic testing has become increasingly valuable in pediatric hepatology and is being integrated into the diagnostic workflow for many liver conditions. For adult patients, instead, access to genetic tests is more limited, likely due to their cost/benefit ratio [6].

However, selected cases do benefit from a genetic assessment, especially in young adults in which a late presentation of an inherited condition [7], as well as liver diseases of unknown origin, may be explained by a genetic condition [8,9].

Historically, genetic analyses for liver diseases were performed with a gene-centered approach which, while reliable, was time-intensive and limited [10]. In recent years, the introduction of Next Generation Sequencing (NGS) techniques has revolutionized the field of genetics, allowing the simultaneous screening of the rapidly increasing number of genes that are being associated with liver phenotypes [11]. To date, the diagnostic yield of NGS-based genetic testing in liver disease is extremely heterogeneous—ranging from 6% to 55% based on the selected population and the sequencing approach [12,13]—and standardized strategies for its optimal clinical application remain lacking.

In this study, we investigate the performance of NGS in a cohort of 203 pediatric and adult patients and exploit results to design optimized age group-based workflows to enhance efficiency, diagnostic rates, and diagnostic precision in different patient subgroups.

## 2. Results

### 2.1. Cohort Description and Demographic Analysis

The cohort included both adults and children, with 71% of patients being <18 years old and a comparable age distribution between males and females (Supplementary Figure S1A,B) and a median age of 8 years (IQR 1-21). One hundred and thirty-three (66.5%) patients were male; family history was positive for 27 patients (13.3%), and the most represented ethnicity was white, which accounted for 84.7% of patients (Supplementary Figure S1C).

The proportion of family history positivity in pediatric and adult patients was comparable (Supplementary Figure S1D); transplanted patients accounted for 27.6% (n = 56) of the whole cohort, and most of them were children (46 out of 56 transplanted patients, Supplementary Figure S1D).

**Figure 1:**
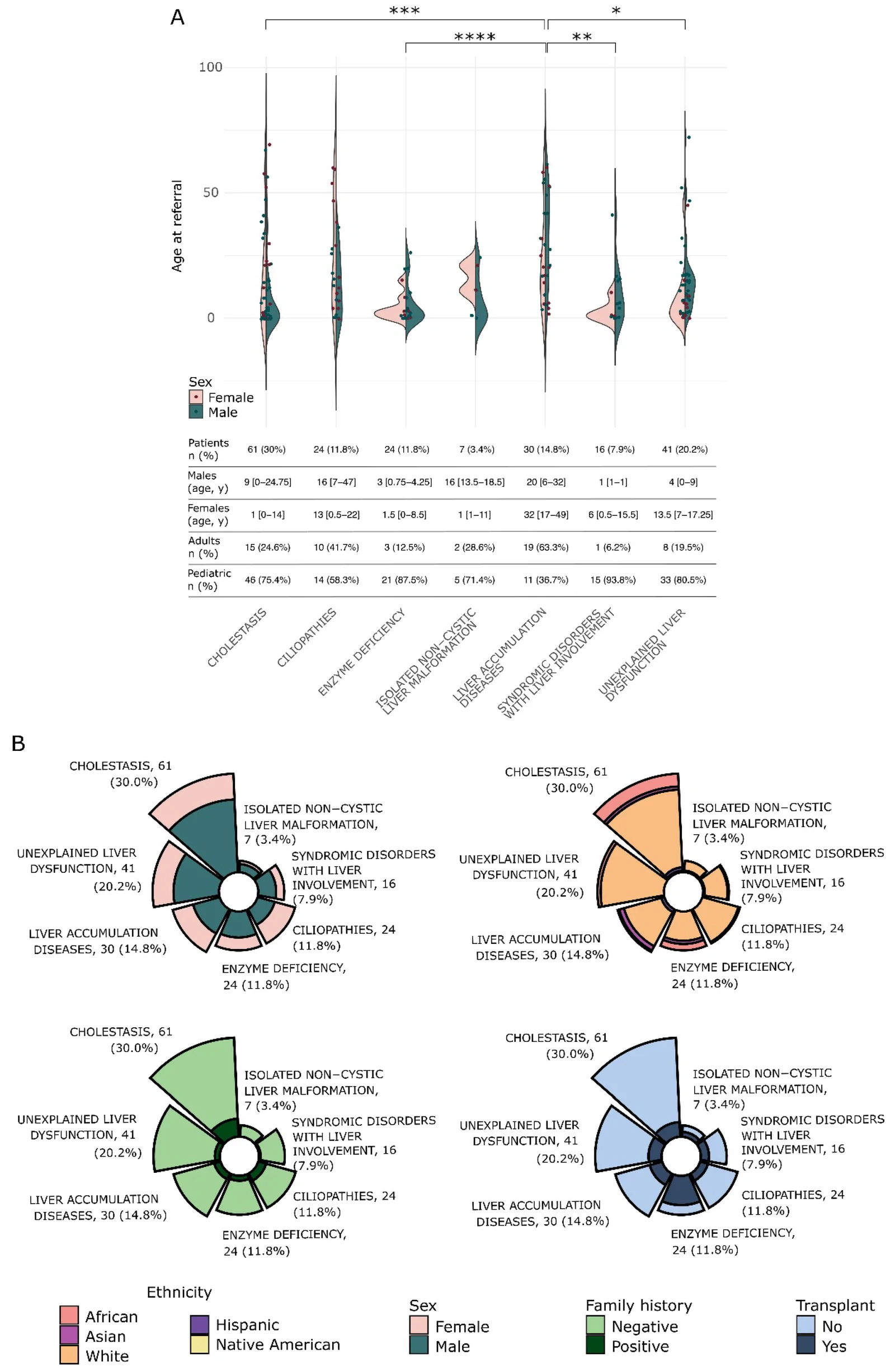
(**A**) Violin plot illustrating the age distribution of female (left side of the violins) and male (right side of the violins) patients across different disease categories. Patient numbers and percentages, as well as median age and interquartile range for each disease and age group, are reported in the table below the violin plot. (**B**) Rose plots showing the distribution of ethnicity, sex, family history, and transplantation status within each disease category. *p*-values are indicated as follows: * < 0.05, ** < 0.01, *** < 0.001, **** < 0.0001.

The categories with more patients were cholestatic liver disorders (n = 61, 30%), unexplained liver dysfunctions (n = 41, 20.2%), and liver accumulation diseases (n = 30, 14.8%), while the others accounted for smaller proportions (Figure 1A). Age distribution in cholestatic disorders, ciliopathies, liver accumulation diseases, and unexplained liver dysfunctions displayed a broad range and included a notable proportion of adult patients (24.6%, 41.7%, 63.3%, and 19.5%, respectively). In contrast, enzymatic deficiencies, isolated non-cystic malformations, and syndromic disorders with hepatic involvement were predominantly pediatric (87.5%, 71.4%, and 93.8%, respectively; Figure 1A).

The proportion of sex, ethnicity, family history positivity, and transplantation status was determined for each macro category (Figure 1B). No significant differences were observed, except for the proportion of transplanted patients within disease categories. Enzyme deficiency showed the highest proportion of transplanted subjects (75%, n = 18), followed by isolated non-cystic liver malformation (42.9%, n = 3) and syndromic disorders with liver involvement (31.2%, n = 5). In contrast, cholestasis and unexplained liver dysfunction showed the lowest proportions of transplanted individuals, at 14.8% (n = 9) and 19.5% (n = 8), respectively, despite their relatively higher total case counts. In detail, the percentage of patients in the enzyme deficiency category was significantly higher, as compared to all other groups (all *p*-values < 0.05 in paired comparisons). This was an expected finding as transplantation is the curative treatment in most cases.

### 2.2. Diagnostic Performance of Genetic Testing Among Age and Diagnostic Categories

**Figure 2:**
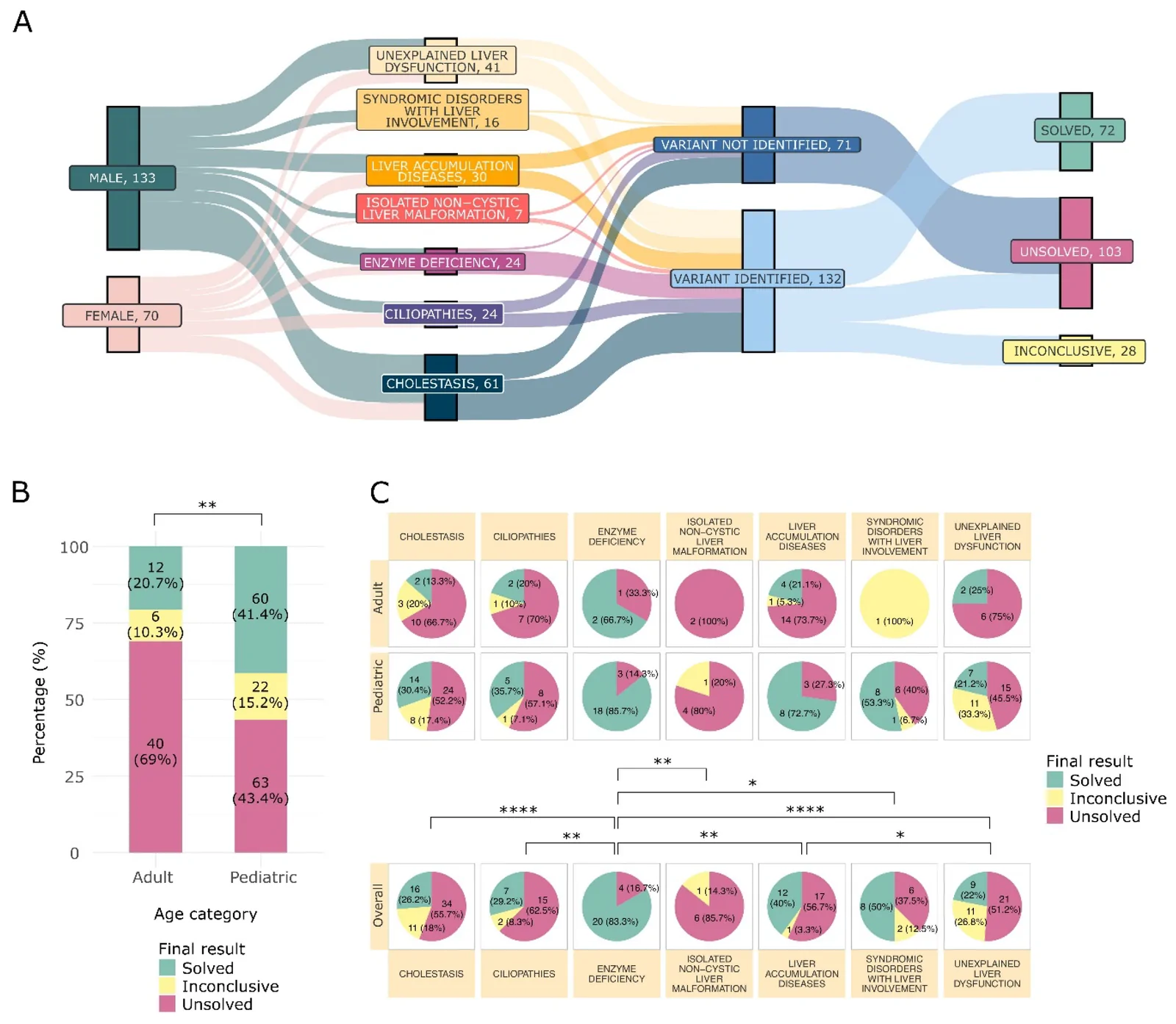
(**A**) Sankey plot summarizing patient sex, clinical diagnosis at the time of referral to our center, identification of a genetic variant in the NGS report (either diagnostic or potentially diagnostic), and the outcome of the genetic consult, including any additional tests or extension of the analysis to other informative family members. (**B**) Bar plot showing the diagnostic rate between age groups. (**C**) Pie charts summarizing the proportion of solved, unsolved, and uncertain cases stratified by disease category and age groups. *p*-values are indicated as follows: * < 0.05, ** < 0.01, **** < 0.0001.

Genetic testing was performed for each recruited patient, with 132 (65%) patients having a positive genetic report, while the remaining 71 (35%) were negative. Out of the genetic reports that contained causative or potentially causative variants, a conclusive diagnosis was obtained for 72 (54.6%) patients, while 34 (24.2%) cases were downgraded to negative (genetic counseling ruled out the role of the reported variants in disease etiology). For 28 (21.2%) patients, the potential causative variant(s) reported could not be further investigated, and genetic counseling could not be concluded, thus leaving the variants with an uncertain clinical significance. Overall, our diagnostic rate was 35.5% (Figure 2A).

Diagnostic yield was also analyzed by splitting the cohort between age groups and among disease categories. Pediatric patients achieved a diagnosis in 41.4% of cases vs. 20.7% of adults (Figure 2B). Among disease categories, enzyme deficiency followed by liver accumulation diseases scored the best diagnostic rates in both age categories (85.7% and 72.7% in children vs. 66.7% and 21.1% in adults, respectively). In the pediatric cohort, syndromic disorders with liver involvement accounted for a good proportion of diagnostic cases (n = 8, 53.3%), while in adults, unexplained liver disorders were successfully diagnosed in 25% of cases (n = 2). The overall most common diagnosed disease categories in our cohort were enzyme deficiency (n = 20, 83.3%), cholestatic disorders (n = 16, 26.2%) and accumulation diseases (n = 12, 40%; Figure 2C).

Diagnostic cases were significantly younger [60 (83.3%) pediatric patients vs. 12 (16.7%) adult patients] and age distribution in the other categories reflected that of the total cohort, except for liver accumulation diseases in which solved cases had a lower median age in the solved cases as compared to the whole cholestatic liver disorders sub-cohort (11.5 vs. 26, Supplementary Figure S2A).

The majority (56) of solved cases were transplanted, and the majority (16) of patients with a positive family history received a diagnosis (Supplementary Figure S2B).

### 2.3. Genes Involved, Types of Variants and Most Common Diagnoses in Conclusive Cases

Among conclusive cases, 106 variants were reported, 52 (49.1%) being classified as pathogenic (C5), 49 (46.2%) as likely pathogenic (C4) variants, and 5 (4.7%) as predisposing polymorphisms (Supplementary Figure S3A). Most of the variants were missense (n = 59, 55.7%), followed by nonsense (n = 16, 15.1%), frameshift indel (n = 13, 12.3%), splicing variants (n = 10, 9.4%), copy number variants (CNVs) (n = 4, 3.8%) inframe indels (n = 2, 1.9%) and 5′-UTR polymorphisms (n = 2, 1.9%) (Supplementary Figure S3B). Among the missense variants, the most common nucleotide substitution was C > T (Supplementary Figure S3C). Variants of unknown significance (C3-VUS, n = 4) identified by CES and potentially causative of the disease were re-evaluated upon completion of family segregation studies or additional testing. Most variants fell within genes associated with autosomal recessive diseases, with only 5 genes associated with autosomal dominant disorders and 3 with X-linked diseases (Supplementary Figure S3D).

Furthermore, we correlated macro categories, transplantation status, and age group with the genes in which causative variants mapped. In most cases, the diagnosed condition confirmed the original clinical suspicion (Supplementary Figure S3E), and most transplanted cases were diagnosed with either a metabolic or cholestatic disease (Supplementary Figure S3F), which are also the most common diagnosed conditions in children (Supplementary Figure S3G). Adults were most often diagnosed with accumulation disease, cholestatic disorders, or ciliopathies (Supplementary Figure S3G).

**Figure 3:**
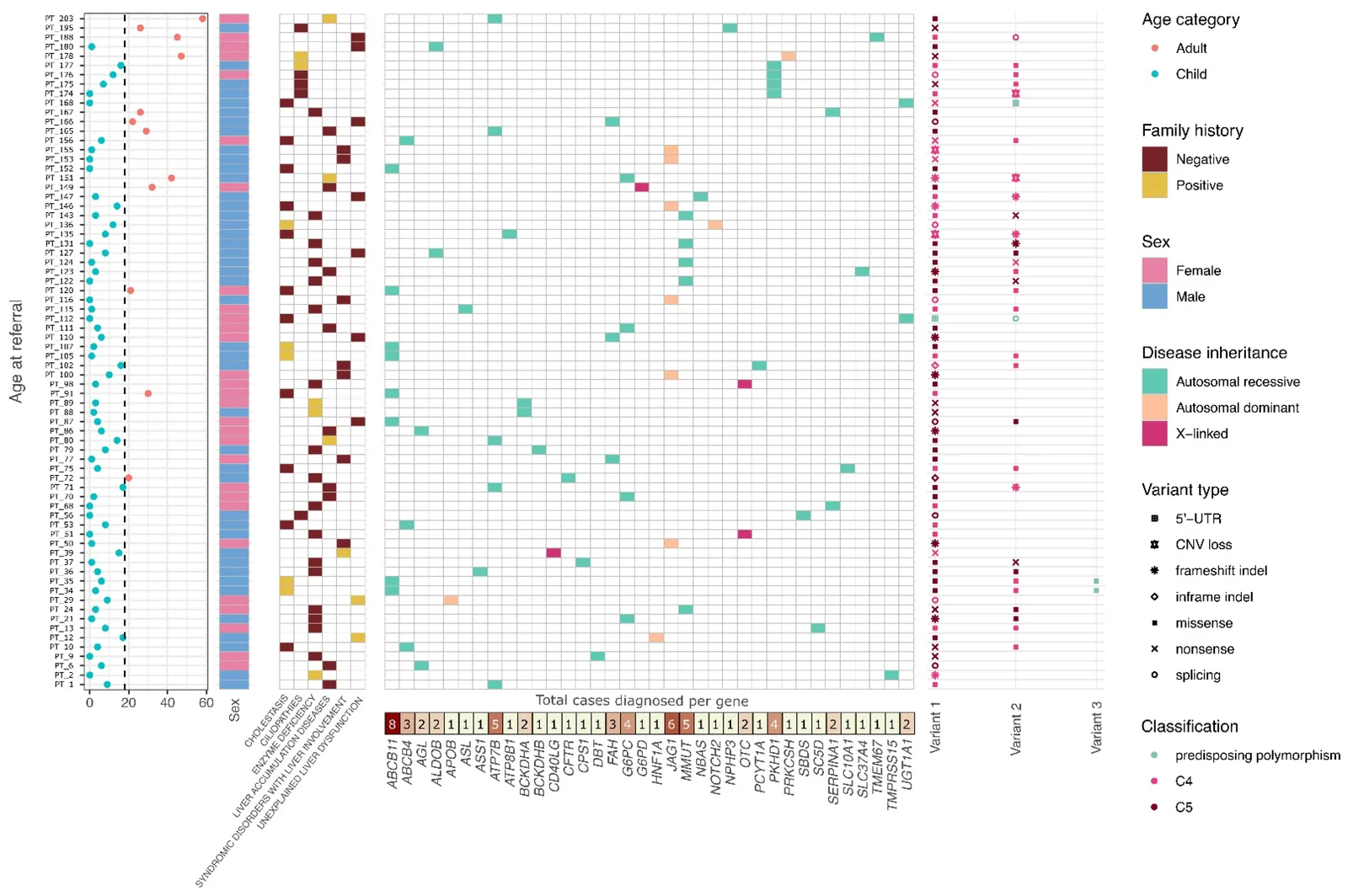
Graphical summary of the characteristics of patients who received a molecular diagnosis. On the left, a scatter plot displays the age of each patient. In the central section, three tile plots represent sex (**left plot**), disease category and family history (**middle plot**), and the causative gene along with the inheritance mode of the associated disease (**right plot**), and the number of cases diagnosed per gene (lower panel). The rightmost plot shows the number, classification (by color), and type (by shape) of each diagnostic variant per patient. When a single variant is associated with an AR condition, its zygosity is homozygous.

Overall, the single most diagnosed condition was PFIC/BRIC [12 cases, 8 of which associated with *ABCB11* (HGNC:42), 3 with *ABCB4* (HGNC:45) and 1 with *ATP8B1* (HGNC:3706) homozygous or compound mutations], followed by Alagille syndrome [7 cases, 6 of which associated with *JAG1* (HGNC:6188) and 1 with *NOTCH2* (HGNC:7882)] and Wilson disease (5 cases; Figure 3). A detailed list of the variants identified in this study is reported in Supplementary Table S2.

### 2.4. Re-Evaluation of Diagnostic Performance After Flowchart Application

**Figure 4:**
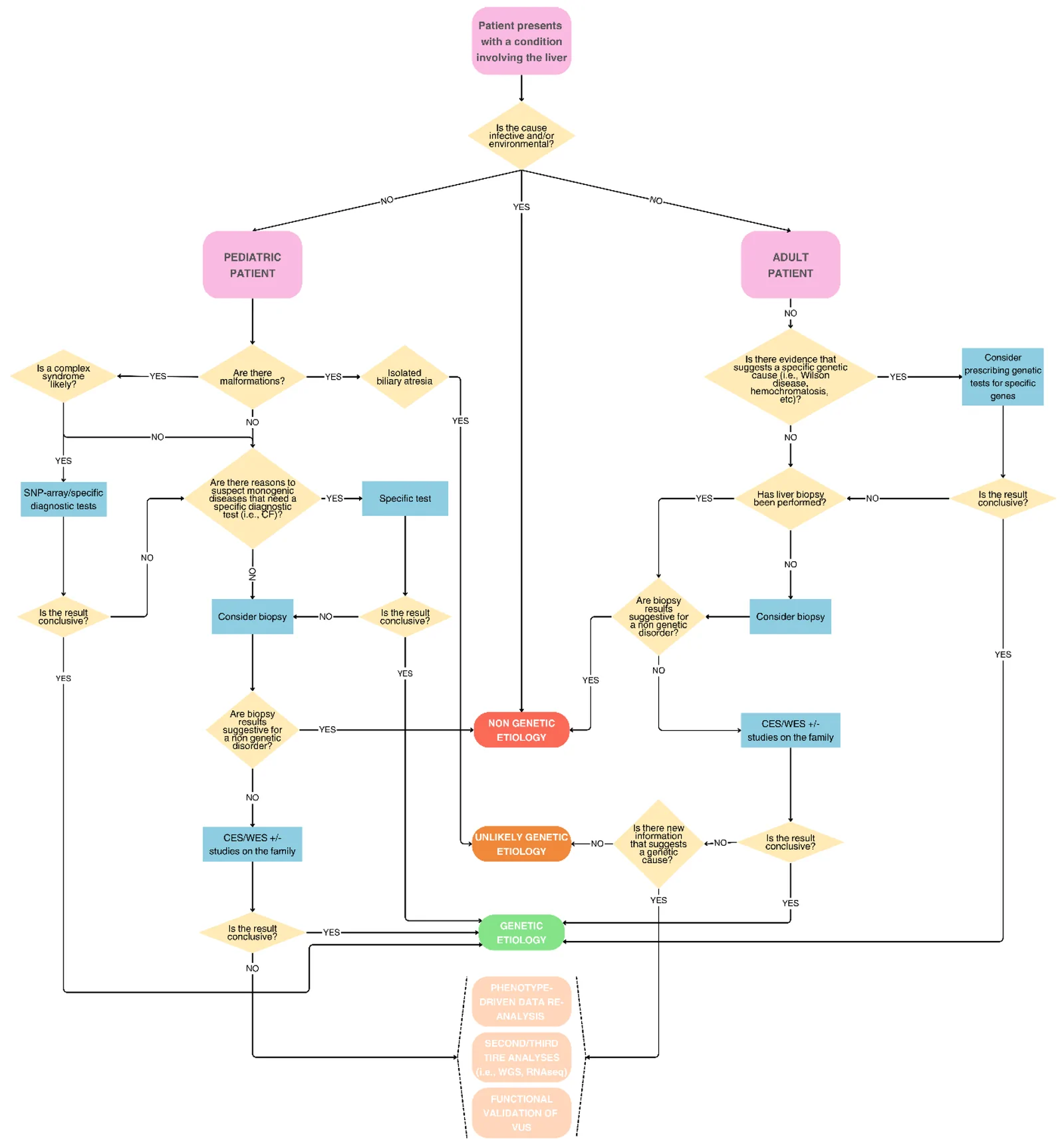
Clinically oriented flowchart for the prescription of genetic testing in pediatric and adult patients. GI: gastroenterologist; SNP: single nucleotide polymorphism; CF: cystic fibrosis; CES: clinical exome sequencing; WES: whole exome sequencing; WGS: whole genome sequencing; RNAseq: RNA sequencing; VUS: variant of unknown significance.

**Figure 5:**
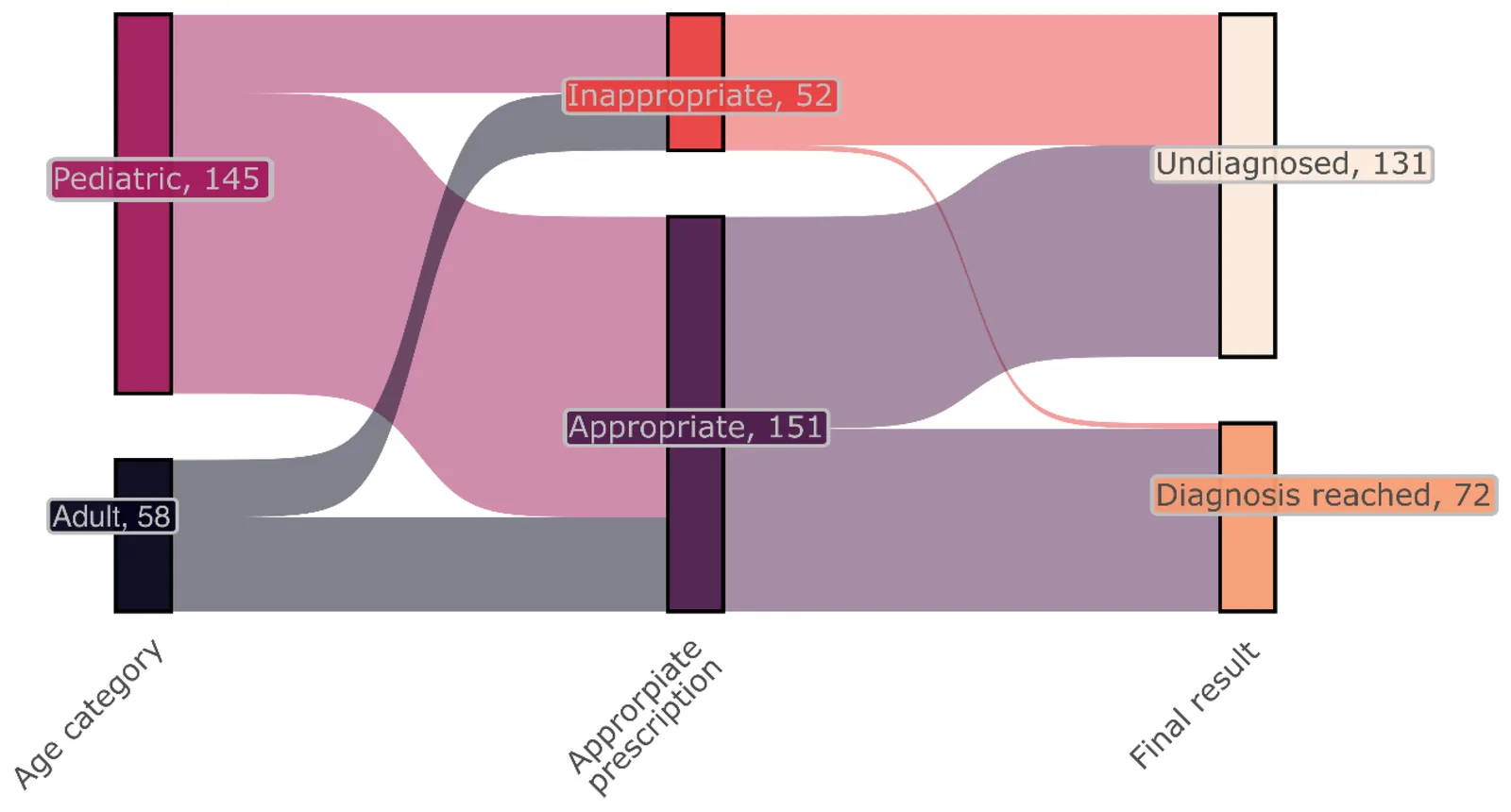
Sankey plot showing the proportion of patients with appropriate prescription stratified by age group. The graph also shows the proportion of appropriate prescriptions that achieved a genetic diagnosis.

As a sufficiently large independent cohort was not available for validation, we assessed the proposed workflow (Figure 4) through internal bootstrap resampling. Each patient was categorized based on the degree of appropriateness of genetic prescription (Figure 5).

After the application of the developed diagnostic flowcharts to the whole cohort, 151 (74.4%) patients still fulfilled the criteria for genetic testing, while for 52 (25.6%) patients the prescription of a genetic test was considered not strictly indicated (Figure 5).

Overall, application of the workflow through internal bootstrap resampling resulted in a retrospective enrichment in diagnostic yield from 35.5% to 46.4%, corresponding to an absolute increase of 10.9 percentage points (BCa 95% CI, 7.5–14.8). The workflow retained 70 of 72 molecular diagnoses, corresponding to a sensitivity of 97.2% (95% CI, 90.4–99.2%) and an NPV of 96.2% (95% CI, 87.0–98.9%, Table 1). The greatest enrichment in diagnostic yield was observed for liver accumulation diseases (+14.5 percentage points), followed by unexplained liver dysfunction (+8.8) and cholestasis (+8.7). No enrichment was observed among patients with syndromic disorders with liver involvement, as all patients in this category were considered appropriate candidates for genetic testing. Similarly, patients with isolated biliary atresia were not considered suitable for workflow-based exclusion from genetic testing, given the potential for an underlying genetic etiology, and performance estimates were therefore not calculated for this subgroup (Table 1).

## 3. Discussion

In this study, we investigate the role of genetic testing in diagnosing acute or subacute liver disorders, assess the performance of CES in a cohort of 203 pediatric and adult patients, and define an optimized diagnostic flowchart to improve diagnostic yield, help clinicians in the decision-making process, and spare resources whenever the test is not appropriate.

Overall, the diagnostic yield reached 35.5%, but differences between children and adults were marked. 41.4% of pediatric patients were successfully diagnosed either with CES alone or with a combination of CES and family segregation studies, which is slightly higher than what is reported in the medical literature [14] and particularly when compared to panel-based NGS sequencing [15]. This is likely due to the continuous update of in silico gene panels that CES allows and to the ongoing discovery of new gene-disease associations.

On the other hand, when considering pediatric cholestatic liver disorders, which accounted for the majority of the cases, the diagnostic yield was comparable to what was achieved by other groups using custom panels [16,17,18]. This is likely due to the molecular basis of the disease being well understood, with most cases being associated with a limited number of genes. For pediatric metabolic disorders, our diagnostic rate was consistent with what was achieved in studies using a very similar approach [13], reflecting the well-established etiology and laboratory features of most metabolic conditions. In cases of unexplained pediatric liver diseases, our diagnostic rate reached 21.2%, proving effective in helping clinical assessment and decision-making. CES also proved effective in suspected syndromic or ciliopathy-related liver disease, yielding diagnoses in 31.8% and 50% of cases. Conditions confirmed included Alagille syndrome, ductal plate malformations, and Shwachman-Diamond syndrome. No diagnoses were made in isolated non-cystic malformations, mainly biliary atresia, which lacks a defined monogenic cause.

The adult cohort in our study showed a drastically different diagnostic yield, with 20.7% of patients reaching a conclusive diagnosis. Although based on smaller sample sizes, the per-category diagnostic rate was generally lower than in the pediatric cohort, apart from unexplained liver dysfunctions. This trend is consistent with the fact that conditions such as ciliopathies, cholestatic disorders, metabolic diseases, and syndromic disorders are typically diagnosed earlier in life. Furthermore, our diagnostic rate for liver diseases of unknown origin appears to be comparable to, or slightly lower than, that reported in previous studies utilizing WES [8,9].

A summary of the most relevant published studies assessing the performance of NGS diagnosis for liver disorders is reported in Table 2.

Overall, this evidence suggests that the application of (clinical) exome sequencing in a diagnostic setting for patients with liver disease should be strongly influenced by both the patient’s age and clinical features.

Based on the results of our cohort analysis, we designed an age-optimized diagnostic workflow whose application resulted in a ~11% retrospective enrichment in the diagnostic rate in the whole cohort, highlighting the importance of a careful and standardized evaluation of the patient’s clinical features.

In pediatric patients, where genetic causes are more likely, our data suggest that exome-based testing should be integrated early into the diagnostic workflow once common non-genetic conditions are excluded. In contrast, for adults with nonspecific liver phenotypes, the diagnostic yield is significantly lower, and the utility of genetic testing should be evaluated carefully, especially in broader clinical settings where NGS may offer limited benefit [12]. In this context, exceptions should be made for subtle and non-self-resolving clinical conditions that can constitute “red flags” and suggest a possible underlying genetic condition.

Regardless of the age, however, genetic testing can help avoid potentially invasive procedures, facilitate treatment decisions (i.e., fructose intolerance [23], PFIC [24]), help in patient stratification and risk assessment [25], and allow informed family planning.

Importantly, genomic findings should not be interpreted in isolation, but integrated with the clinical phenotype, biochemical and histological findings, family history and segregation data, and longitudinal disease course. Therefore, a negative result should always be considered for second- and third-tier testing based on the clinical suspicion and the availability of diagnostic techniques. Such a tiered and iterative strategy may maximize diagnostic yield while limiting unnecessary testing and facilitate the translation of genomic information into individualized clinical care.

Despite the encouraging results, some limitations must be acknowledged. A key limitation is that the proposed diagnostic workflow was developed based on the characteristics of the present cohort and subsequently applied retrospectively to the same population. Therefore, the observed change in the diagnostic yield should be interpreted cautiously, as a potential risk of overfitting and circularity must be considered, and the workflow has not yet been independently validated. This limitation was partially mitigated by a bootstrap resampling approach to assess the stability and uncertainty of the observed estimates. Accordingly, the proposed workflow would benefit from confirmation in an independent, ideally prospective, cohort.

Other limitations are the interpretation of class 3 (VUS) variants, which can be partly overcome by data re-analysis over time [26], and the sequencing approach restricted to a clinical exome (~6700 genes), which misses novel genes, epigenetic changes, regulatory/intronic regions, and may not reliably detect CNVs.

A further limitation of this study is its ethnic bias, as nearly 85% of the patients are of White descent, with other ethnic groups either underrepresented or not represented at all. This imbalance may impact both the diagnostic yield and the spectrum of diseases identified, as suggested by other research groups [16,19]. Although studies involving cohorts primarily or exclusively recruited from Asian countries [14,17] or with a higher proportion of patients of African descent [21] have not demonstrated significantly different results, further research involving larger and more ethnically diverse cohorts is still necessary to better evaluate the influence of ethnicity on genetic liver diseases.

## 4. Materials and Methods

### 4.1. Patients’ Recruitment

The retrospective cohort consists of 203 pediatric (n = 145) and adult (n = 58) patients who were referred for genetic testing to the Immunogenetics and Transplant Biology Service of the Città della Salute e della Scienza Hospital with a liver disease between July 2003 and February 2025 (EC n. 487/2021 of 7th December 2021). All patients included in the study provided written informed consent.

Inclusion criteria were the onset of an acute or subacute liver disorder with features compatible with a potential genetic etiology, the presence of clinical and biochemical alterations suggestive of a genetically determined metabolic disorder, syndromic features with significant or predominant liver involvement and/or unexplained liver dysfunction (i.e., presence of biochemical or radiological abnormalities with negative results upon routine testing). Based on inclusion criteria, patients were assigned a diagnostic macro-category among the following: cholestatic liver disorders, ciliopathies, enzyme deficiencies, isolated non-cystic liver malformations, liver accumulation diseases, syndromic disorders with liver involvement, unexplained liver dysfunction. Data on patients’ ethnicity, sex/gender, and clinical and personal information were collected during counselling. Whenever needed, electronic health records were used to integrate missing information.

Most patients underwent genetic evaluation at the Immunogenetics and Transplant Biology Service of Turin’s University Hospital, where clinical exome sequencing (CES) was performed (sequencing of ~6700 genes with strongly established gene-disease association—vide infra for technical details). Thirty-two additional individuals had been referred to our center earlier (2003–2025), and their genetic results were retrieved from original reports. For these patients, however, genetic testing was not always performed at the time of referral, and many underwent genetic analysis only during a later re-evaluation, once NGS sequencing approaches (CES or gene panels in selected cases) had become standard.

Gene panels to be analyzed were selected according to the clinical presentation and the diagnostic hypothesis, in agreement between the gastroenterologist and the geneticist. Such panels were analyzed in parallel. Whenever deemed necessary, both as a first-tier analysis or in the context of data re-analysis, all variants called in the whole CES were evaluated. After genetic testing, patients underwent genetic counselling and, when appropriate, further analyses were carried out (i.e., CGH-array) as well as family segregation studies.

### 4.2. Next Generation Sequencing

Libraries were prepared using the TruSight One Expanded Sequencing Kit (Illumina, San Diego, CA, USA), covering the coding regions and flanking intronic regions of 6, 794 genes. Genes to be considered for variant identification and prioritization were defined based on clinical suspicion. In silico gene lists were generated by matching data from different databases, correlating genotype to phenotype (OMIM, PanelApp England, ClinGen, Malacards), and data from the literature. A complete list of the genes in each panel is reported in Supplementary Table S1, and an overview of the main disease mechanisms they cover, as well as the total number of genes included and the most relevant and representative ones, is available in Supplementary Figure S4. Sequencing was performed on a NextSeq550 platform (Illumina). Raw data were converted into FASTQ files and then aligned with Enrichment 3.1.0 or DRAGEN Enrichment tools (Illumina) and mapped to the TruSight One Expanded v2.0 manifest using the Homo Sapiens UCSC GRCh37 genome as reference to obtain single nucleotide variants, copy number variants (CNV), and structural variants VCF files. Read alignment and exon coverage of the gene of interest were checked and displayed by Integrative Genomics Viewer—IGV (University of California and the Broad Institute of MIT and Harvard University, Boston; https://software.broadinstitute.org/software/igv/ (accessed on 2023–2025)). Variant prioritization was performed with either Illumina Variant Interpreter or TGex [27] (Geneyx, Herzliya, Israel). CNVs were confirmed using Multiplex Ligation-dependent Probe Amplification (MLPA) and quantitative polymerase chain reaction (q-PCR).

Variant nomenclature was checked with VariantValidator [28] and classification was based on updated ACMG criteria [29,30,31]. Whenever possible, family segregation was performed, and additional analyses were prescribed if deemed appropriate (vide infra). Variant reclassification was actively performed based on the results of additional tests.

### 4.3. Sanger Sequencing

Sanger sequencing of selected variants identified by NGS was performed using standard methods, as reported [32]. Primers were designed with Primer-BLAST (https://www.ncbi.nlm.nih.gov/tools/primer-blast/ (accessed on 2023–2025)), and sequencing was performed on the SeqStudio Genetic Analyzer (Thermofisher, Waltham, MA, USA). Electropherograms were analyzed using the Chromas software version 2.6, freely available at https://technelysium.com.au/wp/chromas/ (accessed on 2023–2025).

### 4.4. Multiplex Ligation-Dependent Probe Amplification (MLPA)

MLPA was performed using commercial kits (MRC Holland, Amsterdam, The Netherlands) following the manufacturer’s protocol. Sequencing was performed on an ABI 3100 Genetic Analyzer (Applied Biosystems, Foster City, CA, USA) and data were analyzed using the Coffalyzer software (MRC Holland, Amsterdam, The Netherlands).

### 4.5. Statistical Analyses and Graphical Rendering

To compare differences in continuous variables between two groups, the Shapiro-Wilk test was applied to assess normality, while Levene’s test was used to evaluate homogeneity of variances. If both assumptions of normality and equal variances were met, an independent samples *t*-test was performed to compare group means. In cases where variances were unequal, Welch’s *t*-test was used. For non-normally distributed data, the Mann-Whitney U test (Wilcoxon rank-sum test) was employed to assess differences in distributions. When comparing continuous variables across three or more groups, data normality was similarly evaluated using the Shapiro-Wilk test, and homogeneity of variances was tested using Levene’s test. One-way analysis of variance (ANOVA) was applied for normally distributed data with equal variances, followed by Tukey’s Honest Significant Difference test for post-hoc comparisons. For non-normally distributed data, the Kruskal-Wallis test was employed, with pairwise Mann-Whitney U tests and Bonferroni correction used for post-hoc analyses. Differences in categorical variables among groups were assessed using the chi-squared test, or Fisher’s exact test when sample sizes were small. Statistical significance was defined as *p* < 0.05, and all analyses and graphical representations were performed using R (version 4.3.3) and the dplyr, ggplot2, scales, tidyverse, ggsankey, stringr, circlize, ggpie, biomaRt packages [33,34,35,36,37,38,39,40,41].

### 4.6. Development and Internal Validation of a Diagnostic Flowchart

Based on the results of this cohort analysis, we evaluated the possible weak points of our approaches, with a particular focus on improving diagnostic rate and optimizing genetic test prescription. Based on this, we developed clinical feature-based diagnostic flowcharts for pediatric and adult patients that could be helpful for gastroenterologists and geneticists in the process of prescribing tests and assessing the final diagnosis. The approach takes into consideration the clinical manifestations (i.e., the presence of malformations and/or associated conditions), the execution of routine testing, as well as the possibility of restricting the diagnostic hypothesis to a specific genetic condition, thus allowing consideration of single-gene testing. The flowchart also highlights that genetic testing should be considered a dynamic and iterative process rather than a single diagnostic step. Accordingly, unresolved cases should periodically undergo reanalysis of existing sequencing data and, if the clinical suspicion remains high, further investigations, including genome sequencing, RNA sequencing, or other targeted functional studies, may be considered, according to the suspected mechanism and their availability.

Given the impossibility of retrieving a novel validation cohort of reasonable size, the proposed diagnostic workflow was retrospectively applied to the study cohort, and its performance was assessed overall and within each diagnostic category. Diagnostic yield was calculated before and after workflow application, and the absolute change in DY and proportion of genetic tests avoided were determined. Uncertainty around these estimates was assessed using nonparametric bootstrap resampling with 5000 replicates, with bias-corrected and accelerated (BCa) 95% confidence intervals. Workflow performance was additionally evaluated using sensitivity, specificity, positive predictive value (PPV), and negative predictive value (NPV), with 95% confidence intervals calculated using the Wilson method. Missed diagnoses were reported as the number and proportion of genetically diagnosed patients who would not have been selected for testing by the workflow. All analyses were performed in R (version 4.3.3).

## Figures and Tables

**Table 1:** Retrospective performance of the proposed genetic testing workflow. Diagnostic yield and workflow performance are reported overall and by diagnostic category. Values in parentheses represent 95% confidence intervals, calculated using BCa bootstrap resampling with 5000 replicates for diagnostic yield before and after workflow application, absolute change in diagnostic yield, and tests avoided, and the Wilson method for sensitivity, specificity, PPV, and NPV. Missed diagnoses are reported as n/N (%). BCa, bias-corrected and accelerated; CI, confidence interval; DY, diagnostic yield; PPV, positive predictive value; NPV, negative predictive value.

Category	N	Before Workflow DY	After Workflow DY	Absolute Δ DY	Tests Avoided	Sensitivity	Specificity	PPV	NPV	Missed Diagnoses
Overall	203	35.5% (28.6–41.9%)	46.4% (38.2–54.2%)	10.9% (7.5–14.8%)	25.6% (19.7–31.5%)	97.2% (90.4–99.2%)	38.2% (30.3–46.7%)	46.4% (38.6–54.3%)	96.2% (87.0–98.9%)	2/72 (2.8%)
Liver accumulation diseases	30	40.0% (20.0–56.7%)	54.5% (31.8–73.9%)	14.5% (6.7–28.0%)	26.7% (10.0–40.0%)	100.0% (75.8–100.0%)	44.4% (24.6–66.3%)	54.5% (34.7–73.1%)	100.0% (67.6–100.0%)	0/12 (0.0%)
Enzyme deficiency	24	83.3% (58.3–91.7%)	87.0% (65.2–95.7%)	3.6% (0.0–16.9%)	4.2% (0.0–12.5%)	100.0% (83.9–100.0%)	25.0% (4.6–69.9%)	87.0% (67.9–95.5%)	100.0% (20.7–100.0%)	0/20 (0.0%)
Cholestasis	61	26.2% (14.8–36.1%)	34.9% (21.4–50.0%)	8.7% (3.1–15.5%)	29.5% (18.0–39.3%)	93.8% (71.7–98.9%)	37.8% (25.1–52.4%)	34.9% (22.4–49.8%)	94.4% (74.2–99.0%)	1/16 (6.2%)
Unexplained liver dysfunction	41	22.0% (9.8–34.1%)	30.8% (14.3–50.0%)	8.8% (0.2–18.6%)	36.6% (22.0–48.8%)	88.9% (56.5–98.0%)	43.8% (28.2–60.7%)	30.8% (16.5–50.0%)	93.3% (70.2–98.8%)	1/9 (11.1%)
Syndromic disorders with liver involvement	16	50.0% (18.8–68.8%)	50.0% (18.8–68.8%)	NA	NA	NA	NA	NA	NA	NA
Isolated non-cystic liver malformation	7	0.0% (0.0–0.0%)	NA	NA	NA	NA	NA	NA	NA	NA
Ciliopathies	24	29.2% (8.3–45.8%)	33.3% (14.3–54.5%)	4.2% (0.8–12.5%)	12.5% (0.0–25.0%)	100.0% (64.6–100.0%)	17.6% (6.2–41.0%)	33.3% (17.2–54.6%)	100.0% (43.9–100.0%)	0/7 (0.0%)

**Table 2:** Comparison of diagnostic study design and diagnostic rate between publicly available studies. CES, Clinical Exome Sequencing; ES, Exome Sequencing.

Cohort	Genetic Test	Suspected Disease	Diagnostic Rate	Reference No.
Pediatric	Gene panels	Monogenic liver diseases	17%	[15]
Pediatric	Gene panels	Neonatal/infantile intrahepatic cholestasis	26%	[16]
Pediatric	Gene panels	Neonatal/infantile intrahepatic cholestasis	32.4%	[17]
Pediatric	Gene panels	Neonatal/infantile intrahepatic cholestasis	28.1%	[18]
Pediatric	CES	Inborn errors of metabolism	55%	[13]
Pediatric	ES	Idiopathic liver injury	40%	[19]
Pediatric	ES	Idiopathic liver injury	37%	[20]
Pediatric	ES	Acute liver failure	39%	[21]
Adults	ES	Idiopathic liver injury	26.3%	[8]
Adults	ES	Idiopathic liver injury	33%	[14]
Pediatric and adults	Multiple approaches	Monogenic liver diseases	21.7–48.8%	[14]
Pediatric and adults	ES	Generic liver disorders	6%	[12]
Pediatric and adults	Gene panels	Cholestatic liver disease	~33%	[22]

## Data Availability

Variants have been submitted to ClinVar under the submitter name University Hospital “Città della Salute e della Scienza di Torino”, with individual SCV accession numbers available on request or through the ClinVar database (SUB15431345). Due to ethical and legal privacy constraints, the raw sequencing data cannot be deposited in a public repository. However, these data will be made available to qualified researchers upon reasonable request to the corresponding author, contingent upon verification of the scientific validity and ethical legitimacy of the request and in compliance with applicable data protection regulations.

## Supplementary Materials

The following supporting information can be downloaded at: https://www.mdpi.com/article/10.3390/ijms27177906/s1.
