# Supplementary material for: Stratified Use of Genetic Testing in Liver Disease Improves Diagnostic Yield and Clinical Impact
Source: Int J Mol Sci. 2026 Sep 4;27(17):7906. doi: 10.3390/ijms27177906 (PMC13566628; doi:10.3390/ijms27177906)
Supplement: Supplementary file 1 [file ijms-27-07906-s001.zip › ijms-4517489-supplementary.pdf]

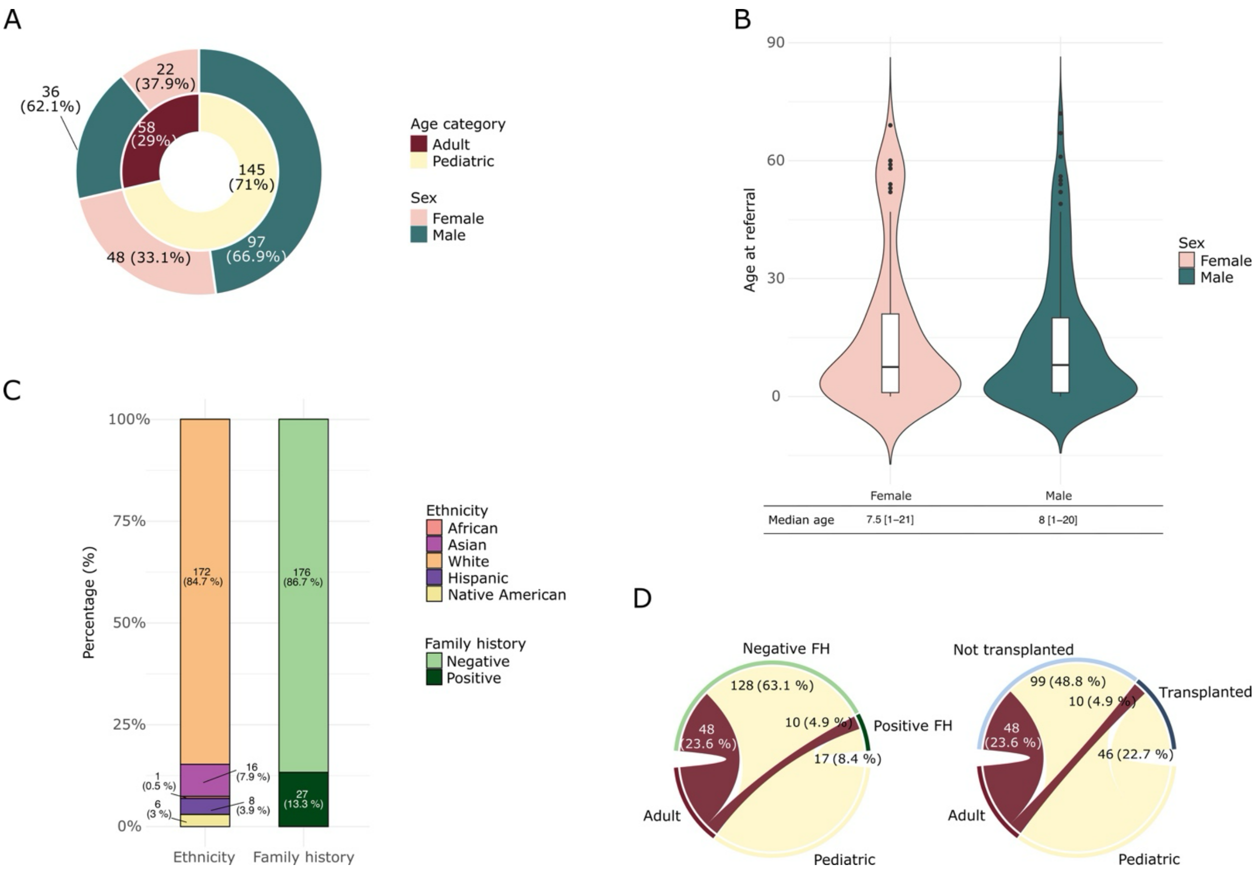

Supplementary Figure S1. A) Doughnut plot showing the percentage of males and females in children and adults. B) Violin plot showing age distribution of patients in the cohort, separated by sex. C) Bar plot displaying the percentage distribution of ethnicity and positive family history across all patients. D) Circular plot depicting the proportion of adults and children with positive and negative family history, as well as the proportion of transplanted patients within each age group.

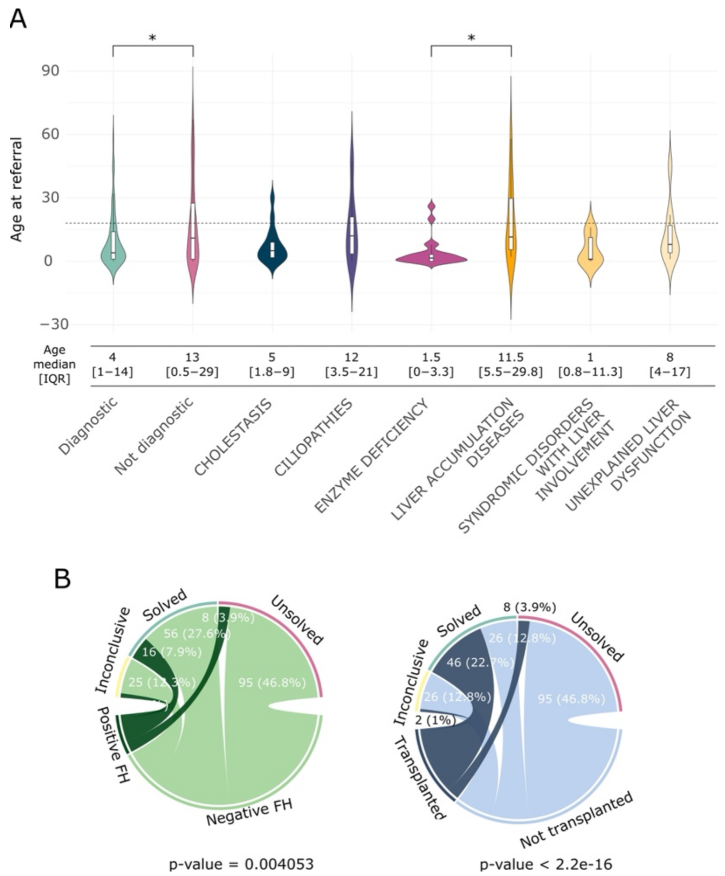

Supplementary Figure S2. A) Violin plots depicting the age distribution of solved and non-diagnostic cases (left side) and the age distribution of solved cases only stratified by disease category. B) Circular plots showing the proportion of solved, unsolved and inconclusive cases with positive or negative family history (left graph) and who underwent or did not undergo transplantation (right graph).

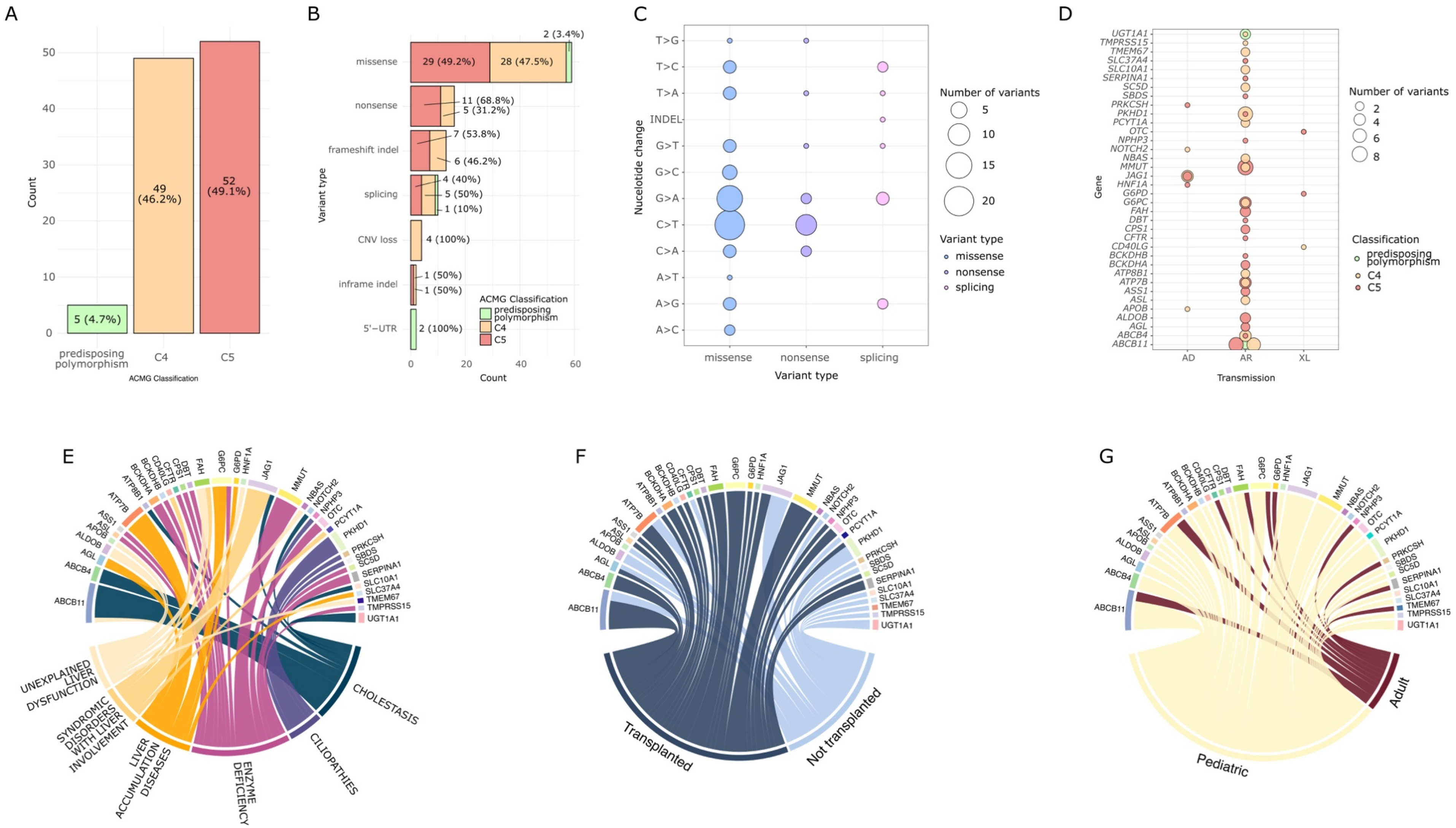

A
Count
5 (4.7%)
49 (46.2%)
52 (49.1%)
predisposing polymorphism
C4
C5
ACMG Classification
B
Variant type
missense
nonsense
frameshift indel
splicing
CNV loss
inframe indel
5'−UTR
29 (49.2%)
28 (47.5%)
2 (3.4%)
11 (68.8%)
5 (31.2%)
7 (53.8%)
6 (46.2%)
4 (40%)
5 (50%)
1 (10%)
4 (100%)
1 (50%)
1 (50%)
2 (100%)
Count
ACMG Classification
predisposing polymorphism
C4
C5
C
Nucelotide change
T>G
T>C
T>A
INDEL
G>T
G>C
G>A
C>T
C>A
A>T
A>G
A>C
missense
nonsense
splicing
Variant type
Number of variants
5
10
15
20
Variant type
missense
nonsense
splicing
D
Gene
UGT1A1
TMPRSS15
TMEM67
SLC37A4
SLC10A1
SERPINA1
SC5D
SBDS
PRKCSH
PKHD1
PCYT1A
OTC
NPHP3
NOTCH2
NBAS
MMUT
JAG1
HNF1A
G6PD
G6PC
FAH
DBT
CPS1
CFTR
CD40LG
BCKDHB
BCKDHA
ATP8B1
ATP7B
ASS1
ASL
APOB
ALDOB
AGL
ABCB4
ABCB11
AD
AR
XL
Transmission
Number of variants
2
4
6
8
Classification
predisposing polymorphism
C4
C5
E
UNEXPLAINED LIVER DYSFUNCTION
SYNDROMIC DISORDERS WITH LIVER INVOLVEMENT
LIVER ACCUMULATION DISEASES
ENZYME DEFICIENCY
CILIOPATHIES
CHOLESTASIS
F
Transplanted
Not transplanted
G
Pediatric
Adult

Supplementary Figure S 3. A) Bar plot displaying the ACMG classification of variants identified in diagnostic cases. B) Graphical representation of the various types of diagnostic variants identified in the cohort. C) Proportion of each nucleotide substitution among the missense, nonsense and splicing variants. D) Bubble plot showing the proportion of variants falling within each gene, their classification and the inheritance mode of the associated disease. E) Circular plot showing the causative genes for each diagnostic macro category. F) Circular plot correlating causative genes with transplantation status. G) Circle plot correlating causative genes with age category.

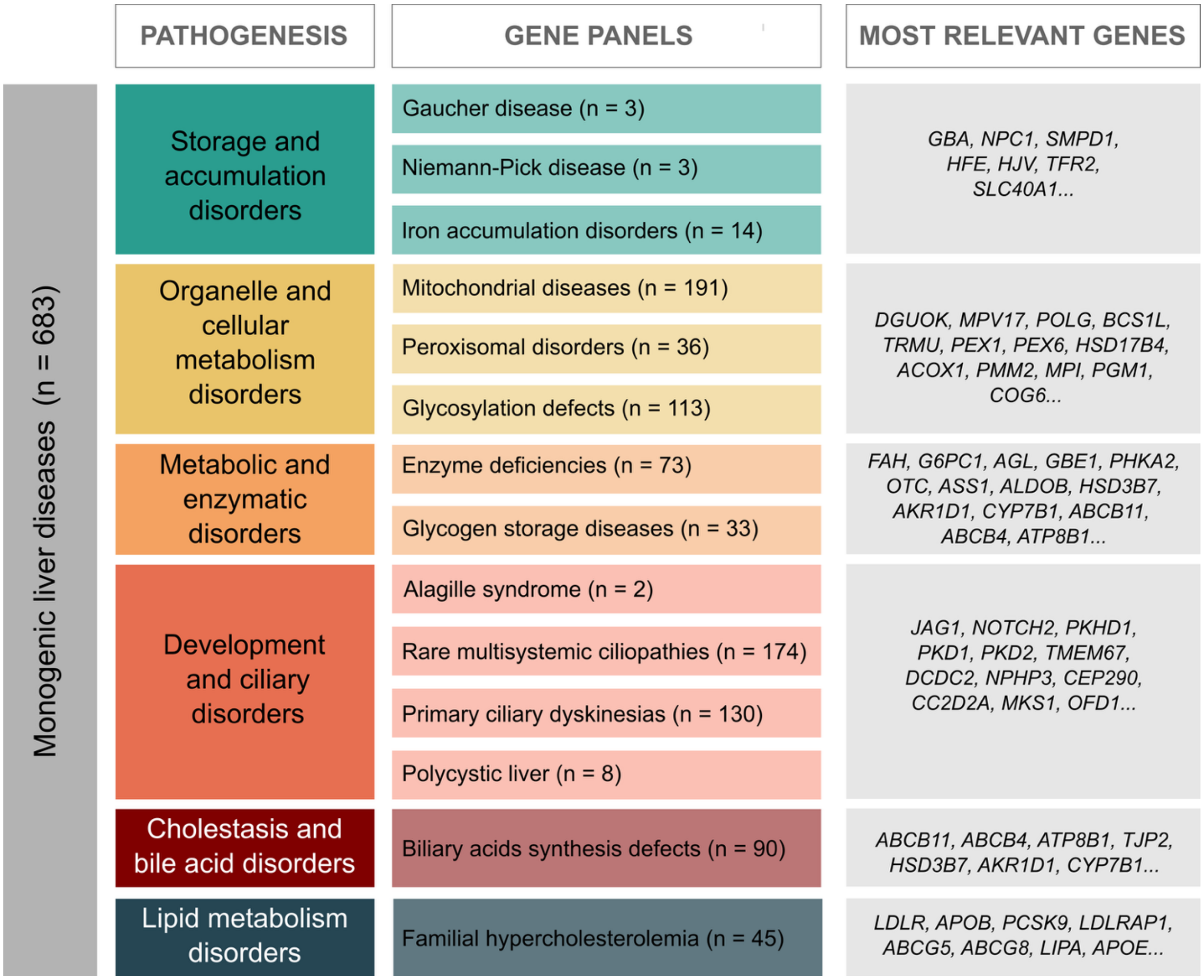

Supplementary Figure S4. Schematic representation of the main mechanisms of disease targeted by each *in silico* panel used for first-tire NGS data analysis. Each gene panel name is followed by the number of genes it includes. On the right of the scheme, the most relevant genes to each pathogenetic category are reported.

Supplementary Table S1. Gene lists included in the *in-silico* gene panels used for genetic testing.

Alagille syndrome

NOTCH2, JAG1

Rare multisystemic ciliopathies

ACVR2B, ADGRV1, AHI1, AIPL1, ALMS1, ANKS6, ARL13B, ARL6, ODAD2, ATXN10, B9D1, B9D2, BBIP1, BBS1, BBS10, BBS12, BBS2, BBS4, BBS5, BBS7, BBS9, CFAP410, CFAP298, C2CD3, PCARE, CPLANE1, C8orf37, CC2D2A, CCDC103, ODAD1, ODAD3, CCDC28B, CCDC39, CCDC40, CCDC65, CCNO, CDH23, CEP120, CEP164, CEP290, CEP41, CEP83, CFAP53, CFC1, CFTR, CLRN1, CRB1, CRB2, CRELD1, CRX, CSPP1, DCDC2, DDX59, DHCR7, DNAAF1, DNAAF2, DNAAF3, DNAAF4, DNAAF5, DNAH1, DNAH11, DNAH5, DNAI1, DNAI2, DNAL1, DRC1, DYNC2H1, EVC, EVC2, EXOC8, FOXH1, GDF1, GLI3, GLIS2, GUCY2D, HNF1B, HYDIN, HYLS1, CILK1, IFT122, IFT140, IFT172, IFT27, IFT43, IFT80, IMPDH1, INVS, IQCB1, KCNJ13, KIF14, KIF7, LBR, LCA5, LEFTY2, LRAT, LRRC6, LZTFL1, MKKS, MKS1, MUC1, MYO7A, NEK1, NEK8, NKX2-5, NME8, NODAL, NPHP1, NPHP3, NPHP4, OCRL, OFD1, PCDH15, PDE6D, PIBF1, PKD1, PKD2, PKHD1, PMM2, POC1A, POC1B, PRKCSH, RD3, RDH12, RPE65, RPGR, RPGRIP1, RPGRIP1L, RSPH1, RSPH4A, RSPH9, SBDS, SCLT1, SCNN1A, SCNN1B, SCNN1G, SDCCAG8, SEC63, SPAG1, SPATA7, SUFU, TCTN1, TCTN2, TCTN3, TMEM138, TMEM216, TMEM231, TMEM237, TMEM67, TOPORS, TRAF3IP1, TRIM32, TSC1, TSC2, TTBK2, TTC21B, TTC8, TULP1, UMOD, USH1C, USH1G, USH2A, VHL, VPS13B, WDPCP, WDR19, DYNC2I2, WDR35, DYNC2I1, WHRN, XPNPEP3, ZIC3, ZMYND10, ZNF423, ZSWIM6

Enzyme deficiencies

ABCC2, ABCD3, ADK, AHI1, AKR1D1, AMACR, ARL13B, ARL3, ASL, ASS1, B9D2, BAAT, C2CD3, CPLANE1, CBY1, CC2D2A, CCBE1, CEP120, CEP290, CEP41, CFTR, CLDN1, CPS1, CSPP1, CYP27A1, CYP7B1, DCDC2, DDX59, DGUOK, DHCR7, EHHADH, FAH, FAT4, HNF1A, HNF1B, HPD, HSD17B4, HSD3B7, CILK1, KIF7, LAMA1, LRPPRC, MKS1, MMUT, NPHP1, OFD1, OTC, PC, PCCA, PCCB, PDE6D, PIBF1, RHAG, RPGRIP1L, SERPINA1, SLC10A2, SLC25A13, SLC27A5, SLCO1B1, SLCO1B3, SUFU, TAT, TCTN1, TCTN2, TCTN3, TMEM138, TMEM216, TMEM231, TMEM237, TMEM67, UGT1A (UGT1A1), UTP4, VIL1

Biliary acids synthesis defects

ABCB11, ABCB4, ABCC12, ABCC2, ABCD3, ADK, AKR1D1, ALDOB, AMACR, AP1S1, ASL, ASS1, ATP7A, ATP7B, ATP8B1, BAAT, BCS1L, CC2D2A, CFTR, CLDN1, COG7, CYP27A1, CYP7B1, DCDC2, DGUOK, DHCR7, FAH, GALE, GALK1, GALT, GBA, GBE1, GFM1, HADHA, HNF1B, HSD17B4, HSD3B7, JAG1, LIPA, MPI, MPV17, MVK, MYO5B, NBAS, NOTCH2, NPC1, NPC2, NPHP3, NR1H4, OTC, PEX1, PEX10, PEX12, PEX13, PEX14, PEX16, PEX19, PEX2, PEX26, PEX3, PEX6, PKHD1, POLG, RINT1, SEMA7A, SERPINA1, SLC10A1, SLC10A2, SLC25A13, SLC27A5, SLC30A10, SLC33A1, SLCO1A2, SMPD1, STX11, STXBP2, TALDO1, TJP2, TRMU, UGT1A, UNC13D, USP53, UTP4, VIPAS39, VPS33B, YARS1, POMC, PRF1, SLC51A, KMT2D

Glycogen storage diseases

AGL, ALDOA, ALDOB, ENO3, EPM2A, FBP1, G6PC1, GAA, GBE1, GYG1, GYS1, GYS2, LAMP2, LDHA, NHLRC1, PC, PCK1, PCK2, PFKM, PGAM2, PGK1, PGM1, PHKA1, PHKA2, PHKB, PHKG2, PRKAG2, PRKAG3, PYGL, PYGM, RBCK1, SLC2A2, SLC37A4

Peroxisomal disorders

ABCD1, ACOX1, AGK, AGPS, AGXT, AMACR, ARSL, CAT, DNM1L, DYM, EBP, FAR1, GNPAT, GRHPR, HOGA1, HSD17B4, IDH1, NSDHL, PEX1, PEX10, PEX11B, PEX12, PEX13, PEX14, PEX16, PEX19, PEX2, PEX26, PEX3, PEX5, PEX6, PEX7, PHYH, SCP2, TRIM37, ACBD5

Iron accumulation disorders

ALAS2, BMP2, CP, FTH1, FTL, HAMP, HFE, HJV, SLC11A2, SLC40A1, STEAP3, TF, TFR2, TMPRSS6

Glycosylation defects

A4GALT, ALG1, ALG11, ALG12, ALG13, ALG14, ALG2, ALG3, ALG5, ALG6, ALG8, ALG9, ARV1, ATP6AP2, ATP6V0A2, B3GALNT2, B3GALT6, B4GALNT1, B4GALT1, B4GALT7, C1GALT1C1, CHST14, CHST3, CHST6, CHSY1, COG1, COG2, COG4, COG5, COG6, COG7, COG8, CSGALNACT1, DDOST, DHDDS, DOLK, DPAGT1, DPM1, DPM2, DPM3, EOGT, EXT1, EXT2, EXTL3, FKRP, FKTN, FUT8, G6PC3, GALNT2, GALNT3, GFPT1, GMPPA, GMPPB, GNE, GORAB, CRPPA, LARGE1, LFNG, MAGT1, MAN1B1, MGAT1, MGAT2, MOGS, MPDU1, MPI, NGLY1, OGT, PGAP1, PGAP2, PGAP3, PGM1, PGM3, PIGA, PIGL, PIGM, PIGN, PIGO, PIGT, PIGV, PIGW, PMM2, POGLUT1, POMGNT1, POMGNT2, POMK, POMT1, POMT2, RFT1, SEC23B, SLC26A2, SLC35A1, SLC35A2, SLC35A3, SLC35C1, SLC35D1, SLC37A4, SRD5A3, SSR4, ST3GAL3, ST3GAL5, STT3A, STT3B, TMEM165, RXYLT1, TRAPPC11, TRAPPC9, TRIP11, TUSC3, VMA21, B3GAT3, B3GLCT, XYLT1, XYLT2

Primary ciliary dyskinesias

ODAD2, ACVR2B, ADGRV1, AGPAT2, AHI1, AIPL1, ARL13B, ARL6, B9D1, B9D2, BBS1, BBS10, BBS12, BBS2, BBS4, BBS5, BBS7, BBS9, CFAP298, CCDC103, ODAD1, ODAD3, CCDC39, CCDC40, CCDC65, CCNO, CPLANE1, CC2D2A, CCDC28B, CDH23, CEP164, CEP290, CEP41, CFTR, CLRN1, CRB1, CRELD1, CRX, DNAH1, DYNC2H1, DNAAF1, DNAAF2, DNAAF3, DNAAF4, DNAAF5, DNAH11, DNAH5, DNAI1, DNAI2, DNAL1, DRC1, EVC, EVC2, FOXH1, GAS2L2, GDF1, GLIS2, GUCY2D, HYLS1, HYDIN, IFT43, IFT80, IMPDH1, INVS, IQCB1, KIF7, LRRC6, LZTFL1, KCNJ13, LCA5, LEFTY2, LRAT, MKKS, MKS1, MYO7A, NEK1, NEK8, NKX2-5, NME8, NODAL, NPHP1, NPHP3, NPHP4, OFD1, PCDH15, PKD2, PKHD1, PCARE, RD3, RDH12, RPE65, RPGRIP1, RPGRIP1L, RPGR, RSPH1, RSPH4A, RSPH9, SPAG1, SCNN1A, SCNN1B, SCNN1G, SDCCAG8, SPATA7, TCTN1, TCTN2, TMEM138, TMEM216, TMEM231, TMEM237, TMEM67, TOPORS, TRIM32, TSC1, TSC2, TTC21B, TTC8, TULP1, UMOD, USH1C, USH1G, USH2A, VHL, WDPCP, WDR19, WDR35, WHRN, XPNPEP3, ZIC3, ZNF423, ZMYND10

Polycystic liver

ALG5, ALG8, LRP5, PKD1, PKD2, PKHD1, PRKCSH, SEC63

Familial hypercholesterolemia

ABCA1, ABCG1, ABCG5, ABCG8, ANGPTL3, ANGPTL4, APOA1, APOA2, APOA5, APOB, APOC2, APOC3, APOE, CEL, CETP, CYP27A1, CYP7A1, DHCR7, EPHX2, FABP2, GHR, GPD1, GPIHBP1, ITIH4, LCAT, LDLR, LDLRAP1, LIPA, LIPC, LIPG, LMF1, LPA, LPL, MTTP, MYLIP, PCSK9, PPARA, PPARG, SAR1B, SCARB1, SLCO1B1, SORT1, SREBF2, USF1, FADS1

Gaucher disease

GBA, PSAP, SCARB2

Mithochondrial diseases

AARS2, ABAT, ABCB7, ACAD9, ACADM, ACADVL, ACAT1, ACO2, AFG3L2, AGK, AIFM1, APTX, ATPAF2, BCS1L, BOLA3, CA5A, CARS2, CHKB, CLPB, COA6, COQ2, COQ4, COQ6, COQ8A, COQ8B, COQ9, COX10, COX14, COX15, COX20, COX5A, COX6A1, COX6B1, COX7B, CYC1, DARS2, DGUOK, DLAT, DLD, DNA2, DNAJC19, DNM1L, EARS2, ECHS1, ELAC2, ETFA, ETFB, ETFDH, ETHE1, FARS2, FASTKD2, FBXL4, FH, FOXRED1, GFER, GFM1, GFM2, GLRX5, GTPBP3, HADHA, HADHB, HARS2, HIBCH, HLCS, HSD17B10, HSPD1, HTRA2, IARS2, IBA57, ISCA2, ISCU, LIAS, LIPT1, LONP1, LRPPRC, LYRM4, LYRM7, MDH2, MGME1, MPC1, MPV17, MRPL3, MRPL44, MRPS2, MRPS22, MTFMT, MTO1, MTPAP, NADK2, NARS2, NDUFA1, NDUFA10, NDUFA12, NDUFA2, NDUFA4, NDUFA9, NDUFAF1, NDUFAF2, NDUFAF3, NDUFAF4, NDUFAF5, NDUFAF6, NDUFB10, NDUFB11, NDUFB3, NDUFB8, NDUFS1, NDUFS2, NDUFS3, NDUFS4, NDUFS6, NDUFS7, NDUFS8, NDUFV1, NDUFV2, NDUFV3, NFS1, NFU1, NUBPL, OPA1, OPA3, PARS2, PC, PCCA, PCCB, PDHA1, PDHB, PDHX, PDP1, PDSS1, PDSS2, PET100, PNPT1, POLG, POLG2, PUS1, RARS2, RRM2B, SARS2, SCO1, SCO2, SDHA, SDHAF1, SDHB, SDHD, SERAC1, SFXN4, SLC19A2, SLC19A3, SLC22A5, SLC25A1, SLC25A13, SLC25A19, SLC25A20, SLC25A3, SLC25A38, SLC25A4, SLC25A46, SPG7, SUCLA2, SUCLG1, SURF1, TACO1, TARS2, TFAM, TIMM8A, TK2, TMEM70, TPK1, TRIT1, TRMU, TRNT1, TSFM, TTC19, TUFM, TWNK, TYMP, UQCRB, UQCRC2, VARS2, XPNPEP3, YARS2, ATP5F1A, ATP5F1E, COA8, FDX2, KARS1, LARS2, MFF, MFN2, RMND1

Niemann-Pick disease

NPC1, NPC2, SMPD1

Monogenic liver diseases

A4GALT, AARS2, ABAT, ABCA1, ABCB11, ABCB4, ABCB7, ABCC12, ABCC2, ABCD1, ABCD3, ABCG1, ABCG5, ABCG8, ACAD9, ACADM, ACADVL, ACAT1, ACBD5, ACO2, ACOX1, ACVR2B, ADGRV1, ADK, AFG3L2, AGK, AGL, AGPAT2, AGPS, AGXT, AHI1, AIFM1, AIPL1, AKR1D1, ALAS2, ALDOA, ALDOB, ALG1, ALG11, ALG12, ALG13, ALG14, ALG2, ALG3, ALG5, ALG6, ALG8, ALG9, ALMS1, AMACR, ANGPTL3, ANGPTL4, ANKS6, AP1S1, APOA1, APOA2, APOA5, APOB, APOC2, APOC3, APOE, APTX, ARL13B, ARL3, ARL6, ARSL, ARV1, ASL, ASS1, ATP5F1A, ATP5F1E, ATP6AP2, ATP6V0A2, ATP7A, ATP7B, ATP8B1, ATPAF2, ATXN10, B3GALNT2, B3GALT6, B3GAT3, B3GLCT, B4GALNT1, B4GALT1, B4GALT7, B9D1, B9D2, BAAT, BBIP1, BBS1, BBS10, BBS12, BBS2, BBS4, BBS5, BBS7, BBS9, BCS1L, BMP2, BOLA3, C1GALT1C1, C2CD3, C8orf37, CA5A, CARS2, CASR, CAT, CBY1, CC2D2A, CCBE1, CCDC103, CCDC28B, CCDC39, CCDC40, CCDC65, CCL2, CCNO, CDH23, CEL, CEP120, CEP164, CEP290, CEP41, CEP83, CETP, CFAP298, CFAP410, CFAP53, CFC1, CFTR, CHKB, CHST14, CHST3, CHST6, CHSY1, CILK1, CLDN1, CLPB, CLRN1, COA6, COA8, COG1, COG2, COG4, COG5, COG6, COG7, COG8, COQ2, COQ4, COQ6, COQ8A, COQ8B, COQ9, COX10, COX14, COX15, COX20, COX5A, COX6A1, COX6B1, COX7B, CP, CPA1, CPLANE1, CPS1, CRB1, CRB2, CRELD1, CRPPA, CRX, CSGALNACT1, CSPP1, CTRC, CTSB, CXCL8, CYC1, CYP27A1, CYP7A1, CYP7B1, DARS2, DCDC2, DDOST, DDX59, DGUOK, DHCR7, DHDDS, DLAT, DLD, DNA2, DNAAF1, DNAAF2, DNAAF3, DNAAF4, DNAAF5, DNAH1, DNAH11, DNAH5, DNAI1, DNAI2, DNAJC19, DNAL1, DNM1L, DOLK, DPAGT1, DPM1, DPM2, DPM3, DRC1, DYM, DYNC2H1, DYNC2I1, DYNC2I2, EARS2, EBP, ECHS1, EHHADH, ELAC2, ENO3, EOGT, EPHX2, EPM2A, ETFA, ETFB, ETFDH, ETHE1, EVC, EVC2, EXOC8, EXT1, EXT2, EXTL3,

FABP2, FADS1, FAH, FAR1, FARS2, FASTKD2, FAT4, FBP1, FBXL4, FDX2, FH, FKRP, FKTN, FOXH1, FOXRED1, FTH1, FTL, FUT8, G6PC1, G6PC3, GAA, GALE, GALK1, GALNT2, GALNT3, GALT, GAS2L2, GBA, GBE1, GDF1, GFER, GFM1, GFM2, GFPT1, GHR, GLI3, GLIS2, GLRX5, GMPPA, GMPPB, GNE, GNPAT, GORAB, GPD1, GPIHBP1, GRHPR, GTPBP3, GUCY2D, GYG1, GYS1, GYS2, HADHA, HADHB, HAMP, HARS2, HFE, HIBCH, HJV, HLCS, HNF1A, HNF1B, HOGA1, HPD, HSD17B10, HSD17B4, HSD3B7, HSPD1, HTRA2, HYDIN, HYLS1, IARS2, IBA57, IDH1, IFT122, IFT140, IFT172, IFT27, IFT43, IFT80, IMPDH1, INVS, IQCB1, ISCA2, ISCU, ITIH4, JAG1, KARS1, KCNJ13, KIF14, KIF7, KMT2D, KRT8, LAMA1, LAMP2, LARGE1, LARS2, LBR, LCA5, LCAT, LDHA, LDLR, LDLRAP1, LEFTY2, LFNG, LIAS, LIPA, LIPC, LIPG, LIPT1, LMF1, LONP1, LPA, LPL, LRAT, LRP5, LRPPRC, LRRC6, LYRM4, LYRM7, LZTFL1, MAGT1, MAN1B1, MDH2, MFF, MFN2, MGAT1, MGAT2, MGME1, MKKS, MKS1, MMUT, MOGS, MPC1, MPDU1, MPI, MPV17, MRPL3, MRPL44, MRPS2, MRPS22, MTFMT, MTO1, MTPAP, MTTP, MUC1, MVK, MYLIP, MYO5B, MYO7A, NADK2, NARS2, NBAS, NDUFA1, NDUFA10, NDUFA12, NDUFA2, NDUFA4, NDUFA9, NDUFAF1, NDUFAF2, NDUFAF3, NDUFAF4, NDUFAF5, NDUFAF6, NDUFB10, NDUFB11, NDUFB3, NDUFB8, NDUFS1, NDUFS2, NDUFS3, NDUFS4, NDUFS6, NDUFS7, NDUFS8, NDUFV1, NDUFV2, NDUFV3, NEK1, NEK8, NFS1, NFU1, NGLY1, NHLRC1, NKX2-5, NME8, NODAL, NOTCH2, NPC1, NPC2, NPHP1, NPHP3, NPHP4, NR1H4, NSDHL, NUBPL, OCRL, ODAD1, ODAD2, ODAD3, OFD1, OGT, OPA1, OPA3, OTC, PARS2, PC, PCARE, PCCA, PCCB, PCDH15, PCK1, PCK2, PCSK9, PDE6D, PDHA1, PDHB, PDHX, PDP1, PDSS1, PDSS2, PET100, PEX1, PEX10, PEX11B, PEX12, PEX13, PEX14, PEX16, PEX19, PEX2, PEX26, PEX3, PEX5, PEX6, PEX7, PFKM, PGAM2, PGAP1, PGAP2, PGAP3, PGK1, PGM1, PGM3, PHKA1, PHKA2, PHKB, PHKG2, PHYH, PIBF1, PIGA, PIGL, PIGM, PIGN, PIGO, PIGT, PIGV, PIGW, PKD1, PKD2, PKHD1, PMM2, PNPT1, POC1A, POC1B, POGLUT1, POLG, POLG2, POMC, POMGNT1, POMGNT2, POMK, POMT1, POMT2, PPARA, PPARG, PRF1, PRKAG2, PRKAG3, PRKCSH, PRSS1, PSAP, PUS1, PYGL, PYGM, RARS2, RBCK1, RD3, RDH12, RFT1, RHAG, RINT1, RMND1, RPE65, RPGR, RPGRIP1, RPGRIP1L, RRM2B, RSPH1, RSPH4A, RSPH9, RXYLT1, SAR1B, SARS2, SBDS, SCARB1, SCARB2, SCLT1, SCNN1A, SCNN1B, SCNN1G, SCO1, SCO2, SCP2, SDCCAG8, SDHA, SDHAF1, SDHB, SDHD, SEC23B, SEC63, SEMA7A, SERAC1, SERPINA1, SFXN4, SLC10A1, SLC10A2, SLC11A2, SLC19A2, SLC19A3, SLC22A5, SLC25A1, SLC25A13, SLC25A19, SLC25A20, SLC25A3, SLC25A38, SLC25A4, SLC25A46, SLC26A2, SLC27A5, SLC2A2, SLC30A10, SLC33A1, SLC35A1, SLC35A2, SLC35A3, SLC35C1, SLC35D1, SLC37A4, SLC40A1, SLC51A, SLCO1A2, SLCO1B1, SLCO1B3, SMPD1, SORT1, SPAG1, SPATA7, SPG7, SPINK1, SRD5A3, SREBF2, SSR4, ST3GAL3, ST3GAL5, STEAP3, STT3A, STT3B, STX11, STXBP2, SUCLA2, SUCLG1, SUFU, SURF1, TACO1, TALDO1, TARS2, TAT, TCTN1, TCTN2, TCTN3, TF, TFAM, TFR2, TIMM8A, TJP2, TK2, TMEM138, TMEM165, TMEM216, TMEM231, TMEM237, TMEM67, TMEM70, TMPRSS6, TOPORS, TPK1, TRAF3IP1, TRAPPC11, TRAPPC9, TRIM32, TRIM37, TRIP11, TRIT1, TRMU, TRNT1, TSC1, TSC2, TSFM, TTBK2, TTC19, TTC21B, TTC8, TUFM, TULP1, TUSC3, TWNK, TYMP, UBR1, UGT1A, UMOD, UNC13D, UQCRB, UQCRC2, USF1, USH1C, USH1G, USH2A, USP53, UTP4, VARS2, VHL, VIL1, VIPAS39, VMA21, VPS13B, VPS33B, WDPCP, WDR19, WDR35, WHRN, XPNPEP3, XYLT1, XYLT2, YARS1, YARS2, ZIC3, ZMYND10, ZNF423, ZSWIM6

Supplementary Table S2. List of diagnostic variants identified in each patient. HOM: homozygous; HET: heterozygous; C5: pathogenic variant; C4: likely pathogenic variant; CNV: copy number variant

Gene	Ref_seq	HGVS	Protein change	Variant type	Zygosity	Classification
ABCB11	NM_003742.4	c.2842C>T	p.Arg948Cys	missense	HET	C5
ABCB11	NM_003742.4	c.3081T>A	p.Ser1027Arg	missense	HET	C4
ABCB11	NM_003742.4	c.1331T>C	p.Val444Ala	missense	HOM	Established risk allele
ABCB11	NM_003742.4	c.2842C>T	p.Arg948Cys	missense	HET	C5
ABCB11	NM_003742.4	c.3081T>A	p.Ser1027Arg	missense	HET	C4
ABCB11	NM_003742.4	c.1331T>C	p.Val444Ala	missense	HOM	Established risk allele
ABCB11	NM_003742.4	c.2178+1G>A	null	splicing	HET	C5
ABCB11	NM_003742.4	c.3628A>C	p.Thr1210Pro	missense	HET	C5
ABCB11	NM_003742.4	c.3422G>T	p.Gly1141Val	missense	HOM	C4
ABCB11	NM_003742.4	c.2629G>A	p.Gly877Arg	missense	HET	C4
ABCB11	NM_003742.4	c.3355A>G	p.Lys1119Glu	missense	HET	C4
ABCB11	NM_003742.4	c.3382C>T	p.Arg1128Cys	missense	HOM	C5
ABCB11	NM_003742.4	c.1708G>A	p.Ala570Thr	missense	HET	C5
ABCB11	NM_003742.4	c.1549C>T	p.Arg517Cys	missense	HET	C4
ABCB11	NM_003742.4	c.2494C>T	p.Arg832Cys	missense	HOM	C5
ABCB4	NM_000443.4	c.475C>T	p.Arg159*	nonsense	HET	C5
ABCB4	NM_000443.4	c.1208A>G	p.Tyr403Cys	missense	HET	C4
ABCB4	NM_000443.4	c.1436C>T	p.Pro479Leu	missense	HOM	C4
ABCB4	NM_000443.4	c.701G>A	p.Trp234*	nonsense	HET	C4
ABCB4	NM_000443.4	c.1744C>T	p.Arg582Trp	missense	HET	C4
AGL	NM_000642.3	c.1736-11A>G	null	splicing	HOM	C5
AGL	NM_000642.3	c.4456del	p.Ser1486Profs*18	frameshift indel	HOM	C5
ALDOB	NM_000035.4	c.524C>A	p.Ala175Asp	missense	HET	C5
ALDOB	NM_000035.4	c.448G>C	p.Ala150Pro	missense	HET	C5

ALDOB	NM_000035.4	c.524C>A	p.Ala175Asp	missense	HOM	C5
APOB	NM_000384.3	c.819-1G>A	null	splicing	HET	C4
ASL	NM_000048.4	c.898G>T	p.Ala300Ser	missense	HET	C4
ASL	NM_000048.4	c.913G>C	p.Gly305Arg	missense	HET	C4
ASS1	NM_054012.4	c.1087C>T	p.Arg363Trp	missense	HET	C5
ASS1	NM_054012.4	c.1168G>C	p.Gly390Arg	missense	HET	C5
ATP7B	NM_000053.4	c.2507G>A	p.Gly836Glu	missense	HOM	C4
ATP7B	NM_000053.4	c.2930C>T	p.Thr977Met	missense	HET	C5
ATP7B	NM_000053.4	c.2304dup	p.Met769fs*26	frameshift indel	HET	C4
ATP7B	NM_000053.4	c.2975C>T	p.Pro992Leu	missense	HOM	C5
ATP7B	NM_000053.4	c.2906G>A	p.Arg969Gln	missense	HOM	C5
ATP7B	NM_000053.4	c.3207C>A	p.His1069Gln	missense	HOM	C5
ATP8B1	NC_000018.9	g.55376306_57363967del	CNV	CNV loss	HET	C4
ATP8B1	NM_001374385.1	c.958_967del	p.Met320Valfs*14	frameshift indel	HET	C4
BCKDHA	NM_000709.4	c.529C>T	p.Gln177*	nonsense	HOM	C5
BCKDHA	NM_000709.4	c.529C>T	p.Gln177*	nonsense	HOM	C5
BCKDHB	NM_183050.4	c.1016C>T	p.Ser339Leu	missense	HOM	C5
CD40LG	NM_000074.3	c.773T>G	p.Leu258*	nonsense	HET	C4
CFTR	NM_000492.4	c.1522_1524del	p.Phe508del	inframe indel	HOM	C5
CPS1	NM_001875.5	c.2440C>T	p.Arg814Trp	missense	HET	C5
CPS1	NM_001875.5	c.3953T>A	p.Leu1318*	nonsense	HET	C5
DBT	NM_001918.5	c.670G>T	p.Glu224*	nonsense	HOM	C5
FAH	NM_000137.4	c.742G>A	p.Gly248Arg	missense	HOM	C5
FAH	NM_000137.4	c.191del	p.Gln64Argfs*28	frameshift indel	HOM	C5
FAH	NM_000137.4	c.554-1G>T	null	splicing	HOM	C5
G6PC1	NM_000151.4	c.79del	p.Gln27Argfs*9	frameshift indel	HET	C5

G6PC1	NM_000151.4	c.247C>T	p.Arg83Cys	missense	HET	C5
G6PC1	NM_000151.4	c.247C>T	p.Arg83Cys	missense	HOM	C5
G6PC1	NM_000151.4	c.247C>T	p.Arg83Cys	missense	HOM	C5
G6PC1	NM_000151.4	c.1004del	p.Leu335fs*24	frameshift indel	HET	C4
G6PC1	NM_000151.4	c.231-11_340+10del	CNV	CNV loss	HET	C4
G6PD	NM_000402.4	c.653C>T	p.Ser218Phe	missense	HET	C5
HNF1A	NM_000545.8	c.815G>A	p.Arg272His	missense	HET	C5
JAG1	NM_000214.3	c.1205dup	p.Gln403Thrfs*13	frameshift indel	HET	C5
JAG1	NM_000214.3	c.1563_1564del	p.Cys522SerfsTer8	frameshift indel	HET	C5
JAG1	NM_000214.3	c.1721-2A>G	null	splicing	HET	C4
JAG1	NM_000214.3	c.22_28del	p.Gly8Profs*5	frameshift indel	HET	C4
JAG1	NM_000214.3	c.2649C>A	p.Cys883*	nonsense	HET	C4
JAG1	NC_000020.10	g.9288451_10654188del	CNV	CNV loss	HET	C4
MMUT	NM_000255.4	c.103C>T	p.Gln35*	nonsense	HET	C5
MMUT	NM_000255.4	c.785G>A	p.Ser262Asn	missense	HET	C5
MMUT	NM_000255.4	c.683G>A	p.Arg228Gln	missense	HET	C5
MMUT	NM_000255.4	c.2200C>T	p.Gln734*	nonsense	HET	C5
MMUT	NM_000255.4	c.655A>T	p.Asn219Tyr	missense	HET	C5
MMUT	NM_000255.4	c.926G>A	p.Trp309*	nonsense	HET	C4
MMUT	NM_000255.4	c.1844C>T	p.Pro615Leu	missense	HET	C5
MMUT	NM_000255.4	c.220dup	p.Ser74Phefs*10	frameshift indel	HET	C5
MMUT	NM_000255.4	c.473G>T	p.Gly158Val	missense	HET	C4
MMUT	NM_000255.4	c.1399C>T	p.Arg467*	nonsense	HET	C5
NBAS	NM_015909.4	c.809G>C	p.Gly270Ala	missense	HET	C4
NBAS	NM_015909.4	c.2926del	p.Ser976Profs*16	frameshift indel	HET	C4
NOTCH2	NM_024408.4	c.6028-5T>A	null	splicing	HET	C4
NPHP3	NM_153240.5	c.3373C>T	p.Arg1125*	nonsense	HOM	C5

OTC	NM_000531.6	c.1015G>A	p.Val339Met	missense	HEM	C4
OTC	NM_000531.6	c.674C>T	p.Pro225Leu	missense	HET	C5
PCYT1A	NM_001312673.2	c.838_840del	p.Glu280del	inframe indel	HET	C4
PCYT1A	NM_001312673.2	c.413A>G	p.His138Arg	missense	HET	C4
PKHD1	NM_138694.4	c.8671C>T	p.Arg2891Cys	missense	HET	C4
PKHD1	NM_138694.4	c.3098-10_3228+12del	CNV	CNV loss	HET	C4
PKHD1	NM_138694.4	c.10174C>T	p.Gln3392*	nonsense	HET	C5
PKHD1	NM_138694.4	c.9107T>G	p.Val3036Gly	missense	HET	C4
PKHD1	NM_138694.4	c.131-1G>A	null	splicing	HET	C4
PKHD1	NM_138694.4	c.6731T>A	p.Leu2244His	missense	HET	C4
PKHD1	NM_138694.4	c.4870C>T	p.Arg1624Trp	missense	HET	C4
PKHD1	NM_138694.4	c.9370C>T	p.His3124Tyr	missense	HET	C4
PRKCSH	NM_001289104.2	c.1290C>G	p.Tyr430*	nonsense	HET	C5
SBDS	NM_016038.4	c.258+2T>C	null	splicing	HOM	C5
SC5D	NM_006918.5	c.86G>A	p.Arg29Gln	missense	HET	C4
SC5D	NM_006918.5	c.632G>A	p.Gly211Asp	missense	HET	C4
SERPINA1	NM_000295.5	c.1096G>A	p.Glu366Lys	missense	HOM	C4
SERPINA1	NM_000295.5	c.1096G>A	p.Glu366Lys	missense	HOM	C5
SLC10A1	NM_003049.4	c.755G>A	p.Arg252His	missense	HET	C4
SLC10A1	NM_003049.4	c.592T>C	p.Cys198Arg	missense	HET	C4
SLC37A4	NM_001467.6	c.1042_1043del	p.Leu348fs*52	frameshift indel	HET	C5
SLC37A4	NM_001467.6	c.902A>C	p.His301Pro	missense	HET	C4
TMEM67	NM_153704.6	c.515G>A	p.Arg172Gln	missense	HET	C4
TMEM67	NM_153704.6	c.1861-2del	null	splicing	HET	C4
TMPRSS15	NM_002772.3	c.1437dup	p.Asn480fs*1	frameshift indel	HOM	C4
UGT1A1	NC_000002.11	g.234668893_234668894dup	null	5'-UTR	HET	Established risk allele

UGT1A4	NM_000463.3	c.997-82T>C	null	splicing	HET	Established risk allele
UGT1A4	NM_000463.3	c.1458C>A	p.Tyr486*	nonsense	HET	C4
UGT1A1	NC_000002.11	g.234668893_234668894dup	null	5'-UTR	HET	Established risk allele
